# Frataxin deficiency unveils cell-context dependent actions of insulin-like growth factor I on neurons

**DOI:** 10.1186/1750-1326-7-51

**Published:** 2012-10-05

**Authors:** Carolina Franco, Silvia Fernández, Ignacio Torres-Alemán

**Affiliations:** 1 Cajal Institute, CSIC, and CIBERNED, Avda Dr Arce 37, 28002, Madrid, Spain

**Keywords:** Friedreich’s ataxia, Frataxin, Insulin-like growth factor 1, Neuroprotection

## Abstract

**Background:**

Friedreich’s ataxia (FRDA) is a neurodegenerative disease caused by deficiency of the mitochondrial iron chaperone frataxin (Fxn). FRDA has no cure, but disease-modifying strategies to increase frataxin are under study. Because insulin-like growth factor I (IGF-I) has therapeutic effects in various types of cerebellar ataxia and exerts protective actions on mitochondrial function, we explored the potential Fxn-stimulating activity of this growth factor on brain cells.

**Results:**

IGF-I normalized frataxin levels in frataxin-deficient neurons and astrocytes through its canonical Akt/mTOR signaling pathway. IGF-I also stimulated frataxin in normal astrocytes but not in normal neurons, whereas IGF-I stimulated the Akt/mTOR pathway in both types of cells. This cell context-dependent action of IGF-I on neurons suggested that the intrinsic regulation of Fxn in neurons is different than in astrocytes. Indeed, neurons express much higher levels of frataxin and are much more sensitive to Fxn deficiency than astrocytes; i.e.: only neurons die in the absence of frataxin. In addition, the half-life of frataxin is shorter in neurons than in astrocytes, while after blockade of the proteasome only neurons responded to IGF-I with an increase in frataxin levels. We also explore a potential therapeutic utility of IGF-I in FRDA-like transgenic mice (YG8R mice) and found that treatment with IGF-I normalized motor coordination in these moderately ataxic mice.

**Conclusion:**

Exposure to IGF-I unveiled a cell-specific regulation of frataxin in neurons as compared to astrocytes. Collectively, these results indicate that IGF-I exerts cell-context neuroprotection in frataxin deficiency that maybe therapeutically effective.

## Background

Insulin-like growth factor I (IGF-I) is an abundant circulating neuroprotective hormone, also synthesized in the brain, that shows a wide variety of actions on brain cells [1]. IGF-I treatment is beneficial for different brain diseases, including various types of cerebellar ataxia in animal models [2-6] and human patients [7]. Whereas general protective actions of IGF-I in the brain are well documented, disease-specific actions of IGF-I, if any, are not well established yet. There are several neurodegenerative conditions where IGF-I dysfunction is probable. For example, there is evidence of specific disturbances in IGF-I signaling in spinocerebellar ataxia 1 and 7 [8]. Two other ataxic diseases with different etiology and pathology, ataxia telangectasia and Friedreich’s ataxia (FRDA), may also show disturbed IGF-I function. Both types of ataxia show cumulative DNA damage. In the case of ataxia telangectasia this is due to impaired DNA repair, [9] while in FRDA excess oxidative stress appears as the main culprit [10]. In turn, DNA damage is known to reduce IGF-I activity [11]. At any rate, these two types of ataxia present insulin resistance [12-14], a condition often associated to IGF-I dysfunction [15].

In the present work we focused on a possible relationship of IGF-I to FRDA, the main type of human inherited ataxia. FRDA is associated to mitochondrial dysfunction due to reduced frataxin levels, a mitochondrial iron chaperone involved in the metabolism of Fe-S clusters [10]. Although we previously postulated that IGF-I administration may exert a beneficial effect in all types of ataxia through its wide neuroprotective activities [16], in the case of FRDA a disease-modifying effect of IGF-I may be envisaged if it improves frataxin function by modulating mitochondrial activity. The latter is theoretically supported by the fact that brain mitochondria are targeted by IGF-I [17] and that IGF-I enhances mitochondrial activity [18,19]. The present work indicates that IGF-I stimulates frataxin levels in a cell-context fashion and is able to restore motor function in a mouse model of FRDA with moderate ataxia.

## Results

### IGF-I modulates frataxin in a cell-context fashion

**Figure 1:**
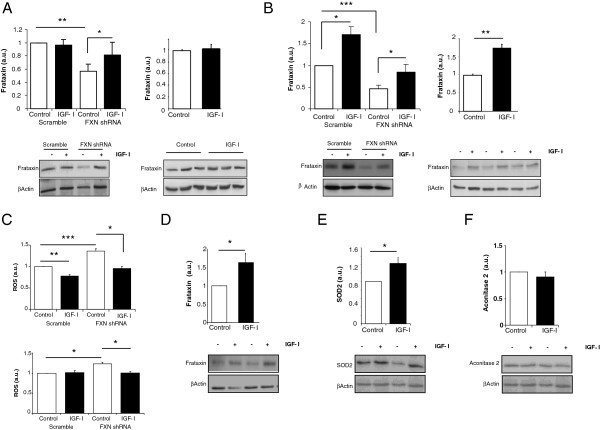
**IGF-I stimulates frataxin in a cell-context fashion. A**, Treatment with IGF-I (100 nM, 24 hours) increased frataxin levels in frataxin-deficient (right) but not in normal neurons (left). **B**, IGF-I stimulated frataxin both in frataxin-deficient and normal astrocytes. **C**, IGF-I normalized levels of reactive oxygen species (ROS) in frataxin-deficient astrocytes (upper panel) and neurons (lower panel). **D**, IGF-I stimulated frataxin content in cardiomyocytes. **E**, Levels of SOD2 are increased by IGF-I in wild type astrocytes. **F**, IGF-I did not alter aconitase levels in astrocytes. Western blots were performed to measure the levels of frataxin, SOD2 and aconitase, and protein load normalized against β-actin. Representative blots are shown; the upper blots correspond to frataxin (**A**, **B**, **D**), SOD2 (**E**) or aconitase (**F**) and the lower blots to β-actin (n=3-6, independent experiments); *p<0.05; **p<0.01; ***p<0.001.

In FRDA patients, frataxin deficiency affects all cells in the body although the disease manifests mostly as a neurological illness. Hence, we determined possible effects of IGF-I on frataxin-deficient neurons and astrocytes, the two most abundant cell types in the brain. Both neurons and astrocytes made deficient in frataxin after viral-mediated transduction of frataxin shRNA showed recovered frataxin levels after 24 hours of treatment with IGF-I (Figure 1A,B). An unspecific effect of IGF-I on RNA interference was ruled out using calcineurin siRNA in astrocytes. In this case IGF-I did not affect the siRNA-induced calcineurin decrease (not shown). IGF-I also normalized reactive oxygen species (ROS) levels in frataxin-deficient astrocytes or neurons (Figure 1C). Notably, while IGF-I did not raise frataxin levels in normal cerebellar (Figure 1A, righ panel) or dorsal root ganglia neurons (not shown), it stimulated frataxin in normal astrocytes (Figure 1B, right panel). To ascertain whether IGF-I raises frataxin in other normal cells, we determined its effects on cardiomyocytes, another cell type affected in FRDA patients. As shown in Figure 1D, IGF-I significantly increased levels of frataxin in cardiomyocites. Finally, we determined whether IGF-I specifically affects frataxin or other mitochondrial proteins are also affected by this growth factor. As shown in Figure 1E, IGF-I increased SOD2, another mitochondrial protein with antioxidant activity. However, another mitochondrial protein such as aconitase, involved in oxidative metabolism, was not altered by IGF-I (Figure 1F).

**Figure 2:**
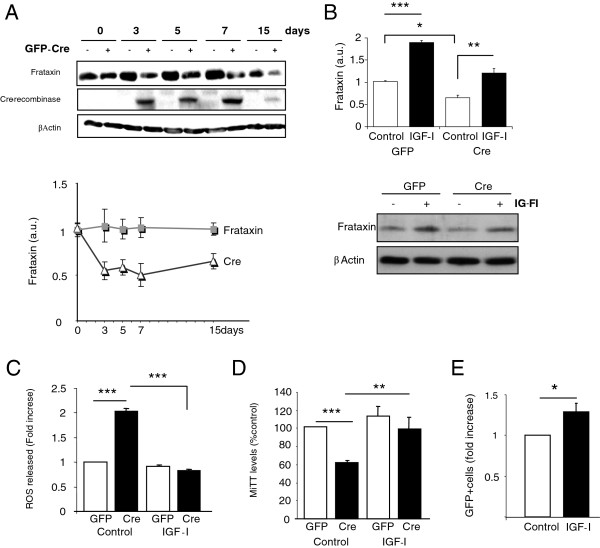
**Beneficial actions of IGF-I under severe frataxin deficiency. A**, Transfection of floxed-Fxn astrocytes with GFP-Cre decreased frataxin levels along time. Representative blot and quantification graphs of frataxin levels along time in culture are shown. **B**, Treatment of Cre-deleted astrocytes with 100nM IGF-I for 24 hours increased frataxin levels. Representative blots are shown. **C**, Increased levels of ROS in frataxin-deficient cultures were normalized by IGF-I. **D**, Disturbed mitochondrial activity due to Fxn deficiency was also corrected by IGF-I as determined by normalization of MTT levels. **E**, Astrocytes promote survival of frataxin-deficient neurons when 100nM of IGF-I is added to the co-cultures for 24 hours. GFP^+^ (frataxin-deleted)-neurons were counted (n=4-5, independent experiments), *p<0.05, **p<0.01 and ***p<0.001.

While ablation of frataxin in mice is lethal [20], we wanted to determine whether the survival of neurons and astrocytes is also fully dependant on frataxin and whether IGF-I exerts any protective effect in frataxin-deleted cells. We ablated Fxn by expressing GFP-tagged Cre recombinase in Fxn gene-floxed neurons and astrocytes. IGF-I could not rescue neurons because in the absence of Fxn they died very rapidly (within less than 48 hours) even though only ≤40% of the neurons were transfected with Cre (GFP^+^). Therefore, a non-cell autonomous toxic effect appears to kill the rest of the neurons with normal levels of frataxin. Conversely, in Fxn-floxed astrocytes, expression of Cre led to a pronounced decrease in frataxin levels as expected (Figure 2A), but the cells remained alive and levels of frataxin increased in response to IGF-I (Figure 2B). Thus, a clear difference exists between astrocytes and neurons in respect to their resilience to the lack of frataxin. Further, as Cre-mediated ablation of floxed genes is irreversible, IGF-I must increase frataxin only in non-deleted cells, as already seen in non-transfected wild type astrocytes (Figure 1B). As seen in Fxn shRNA-transfected astrocytes (Figure 1C), IGF-I also normalized ROS levels in Cre-deleted, Fxn-floxed astrocyte cultures (Figure 2C). Furthermore, astrocyte metabolism measured with the MTT assay, a marker of mitochondrial activity [21], was also normalized by IGF-I under these conditions (Figure 2D). Again, these results suggest a non-cell autonomous effect of frataxin. In this case, frataxin was raised by IGF-I only in intact astrocytes, but protected those without frataxin as well. Because astrocytes are important contributors to neuronal health we then determined whether protection of astrocytes by IGF-I could impact on neurons. We treated with IGF-I Cre-transfected (GFP^+^), Fxn-floxed neurons (unable to survive when cultured alone) co-cultured with wild type astrocytes. The mere presence of astrocytes allowed neurons without frataxin (GFP^+^) to survive, and treatment with IGF-I modestly, but significantly increased their number after 24 hours in co-culture (Figure 2E).

**Figure 3:**
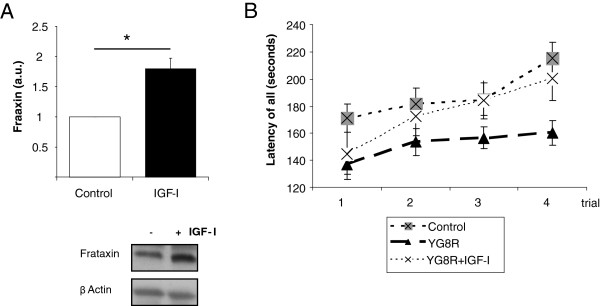
**Therapeutic utility of IGF-I. A**, IGF-I (100 nM) treatment for 24 hours stimulates frataxin in human astrocytes (n=3, independent experiments). **B**, One month of treatment of YG8R mice (4–6 months-old) with subcutaneous IGF-I (Alzet minipumps, 50 μg/kg/day) resulted in normalization of motor coordination in the rota-rod (F=10.1; n= 8–30). No effects on body weight were observed in treated mice. *p<0.05.

We then determined whether the neuroprotective effects exerted by IGF-I in vitro could translate into a therapeutic action. First, we analyzed whether IGF-I is active in human cells. We used human astrocytes because murine astrocytes readily responded to IGF-I. Indeed, human astrocytes showed increased Fxn levels after treatment with IGF-I (Figure 3A). Next, we used a mouse model of FRDA to determine the in vivo potential of IGF-I. Albeit with the usual limitations of animal models, YG8R mice bearing the human mutation in a null mouse frataxin background are currently the model that most closely resembles the molecular basis of human FRDA [22]. All available mouse models of FRDA show either a severe ataxic phenotype and die soon after birth, or a modest degree of motor incoordination, which is the case of YG8R mice. We treated YG8R mice with sc IGF-I (50 μg/kg/day, Alzet minipumps) for 1 month and determined motor coordination in the rota-rod test. As shown in Figure 3B, IGF-I restored rota-rod performance to control levels (p<0.01 vs untreated YG8R mice). However, brain frataxin levels were not changed by IGF-I treatment (not shown). Collectively these data support a therapeutic action of IGF-I in FRDA.

### IGF-I modulates frataxin through a PI3K/Akt/mTOR pathway

**Figure 4:**
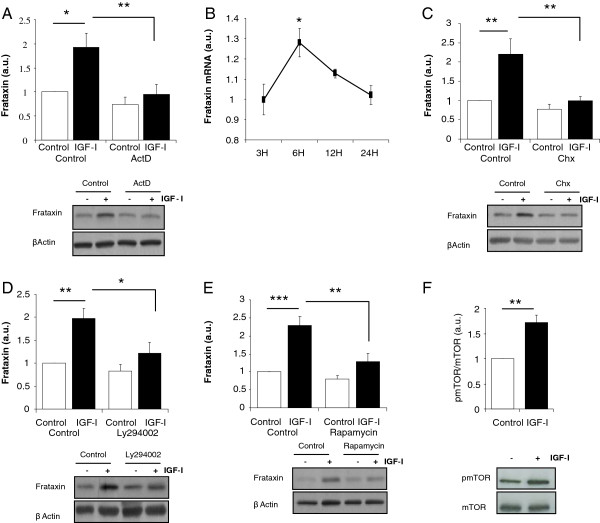
**IGF-I stimulates frataxin in astrocytes through the PI3K/Akt/mTor pathway. A**, Inhibition of mRNA transcription with actinomycin D (ActD, 2 μg/ml) eliminated the stimulatory effect of IGF-I on frataxin levels. **B**, IGF-I significantly stimulates frataxin mRNA 6 hours after its addition to astrocyte cultures returning to basal levels at 24 hours post-addition. **C**, Inhibition of protein synthesis with cycloheximide (Chx, 1 μg/ml) blocked the stimulatory effects of IGF-I on Fxn levels in astrocytes. **D**, Inhibition of PI3 kinase with 100 μM of Ly294002 inhibited the stimulatory action of IGF-I on frataxin levels. **E**, Similarly, inhibition of mTor with rapamycin (100nM) also blocked the positive effect of IGF-I. **F**, IGF-I stimulates phosphorylation of mTOR (pmTOR). Representative blot are shown; the upper blots correspond to frataxin and the lower blots to β-actin (n=4-6, independent experiments). *p<0.05; **p<0.01, and ***p<0.001.

We next analyzed intracellular pathways underlying the stimulatory actions of IGF-I. We first determined whether IGF-I stimulates frataxin expression by inhibiting mRNA transcription with actinomycin D. In the absence of mRNA synthesis, the stimulatory action of IGF-I on frataxin levels in astrocytes was blocked (Figure 4A). Using qPCR we confirmed that synthesis of Fxn mRNA is increased by IGF-I in astrocytes (Figure 4B). Accordingly, inhibition of protein translation with cycloheximide also abrogated the stimulatory action of IGF-I on astrocytes (Figure 4C). Next, among canonical pathways stimulated by IGF-I we found that the PI3K/Akt/mTOR pathway is involved. Inhibition of Akt activation with the PI3kinase inhibitor Ly294002 or of mTOR with rapamycin blocked the stimulatory action of IGF-I (Figure 4D, E). In addition, levels of phosphorylated mTOR, an indirect measurement of its activity status, were also increased after IGF-I (Figure 4F). Blockade of other kinases downstream of the IGF-I receptor such as PKC did not modify the increase in frataxin after IGF-I treatment (not shown).

### Mechanisms underlying cell-context effects of IGF-I on neurons

**Figure 5:**
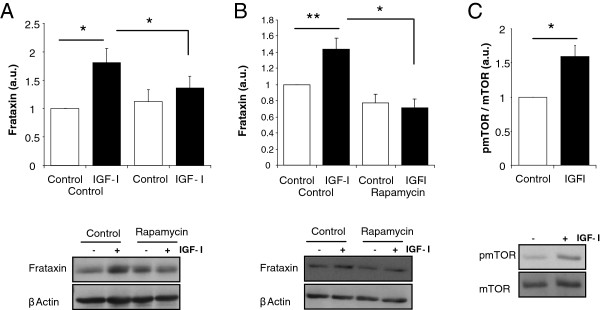
**IGF-I acts through mTOR in frataxin-deficient cells and normal neurons.** IGF-I stimulates frataxin in a rapamycin (100nM) sensitive way in frataxin-deficient astrocytes (**A**) and neurons (**B**). **C**, IGF-I increases phosphorylation of mTOR in neuronal cultures. Representative blot are shown; the upper blots correspond to frataxin and the lower blots to β-actin (n=3-4, independent experiments). *p<0.05; **p<0.01.

**Figure 6:**
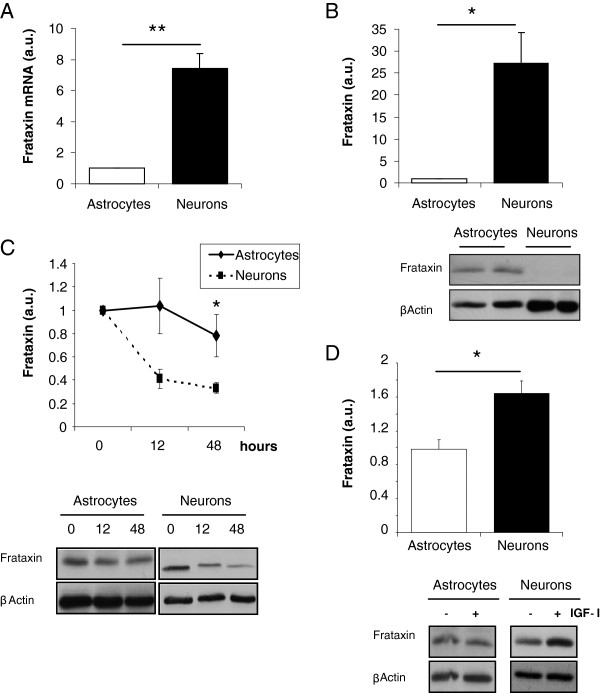
**Differential regulation of frataxin in neurons and astrocytes.** A large difference was appreciated between neurons and astrocytes in levels of frataxin mRNA (**A**) and protein (**B**). **C**, Inhibition of protein synthesis with cycloheximide (1 μg/ml) for 48 hours, unveils a longer half life of frataxin in astrocytes than in neurons (time effect by ANOVA: F=5.32). **D**, Addition of IGF-I (100 nM, 10 hours) to proteasome-inhibited cells (10μM of MG-132) resulted in an increase in frataxin levels in neurons but not in astrocytes. Representative blot are shown for B,C and D; the upper blots correspond to frataxin and the lower blots to β-actin (n=3-4, independent experiments). *p<0.05; **p<0.01.

We then analyzed mechanisms underlying cell-context actions of IGF-I on neurons. We first determined whether mTOR is also involved in the stimulatory effect of IGF-I on frataxin -deficient astrocytes and neurons. Indeed, rapamycin blocked the stimulatory effect of IGF-I on shRNA-transfected astrocytes (Figure 5A), and neurons (Figure 5B). Next, we found that in normal neurons IGF-I also stimulated mTOR phosphorylation (Figure 5C), even though frataxin levels remain unchanged (Figure 1A)*.* Thus, activation of mTOR by IGF-I is necessary but not sufficient to increase frataxin levels in neurons. We then explored potential differences between astrocytes and neurons in an attempt to understand the lack of effect of IGF-I on normal neurons. We found that under basal conditions the levels of frataxin mRNA and protein are very high in neurons as compared to astrocytes (Figure 6A,B). In addition, the half-life of frataxin in neurons was significantly shorter than in astrocytes (Figure 6C). After inhibition of protein synthesis with cycloheximide, levels of frataxin dropped significantly faster in neurons (time effect by ANOVA: F=5.32, p<0.05). As these results indicate that frataxin degradation is faster in neurons than in astrocytes, we inhibited proteasome activity with MG132 in these two types of cells because frataxin has been shown to be degraded through the proteasome [23]. Under these conditions IGF-I was able to stimulate Fxn levels in wild type neurons but not in astrocytes (Figure 6D). IGF-I also increased Fxn in neurons treated with lactacystin, another proteasome inhibitor, albeit more modestly (not shown).

## Discussion

Our results suggest that IGF-I exerts cell context-dependent stimulatory effects on Frataxin levels in neurons. Thus, IGF-I stimulated frataxin in cerebellar neurons only under frataxin deficiency or proteasome inhibition, that is, only under conditions stressful to the cell but not under normal conditions. However, IGF-I stimulated the mTOR pathway both in normal and in frataxin-deficient neurons, regardless of its ultimate effects on frataxin levels; i.e.: the mechanism of action of IGF-I is essentially the same in neurons and astrocytes. Hence, it seems that under normal conditions, IGF-I stimulates frataxin in neurons, but this stimulatory action is masked by a parallel increase in its degradation. Two observations support this notion. First, basal expression of frataxin in neurons is higher than in astrocytes. Probably this reflects a greater dependency of neurons on this mitochondrial chaperone; i.e.: neurons, but not astrocytes, die in the absence of frataxin. At the same time, the half-life of frataxin is much shorter in neurons than in astrocytes. This suggests that proteasome degradation (frataxin has been shown to be degraded through the proteasome [23]), is more active in neurons than in astrocytes. Whether this is specific for frataxin or merely reflects an overall greater proteasome activity in neurons will require further study. Collectively, these data suggest that under basal conditions, frataxin levels in neurons are tightly regulated within a narrower threshold than in astrocytes (and probably other cell types). This is achieved by a balance between higher expression and continued degradation. When this balance is disrupted, such as by RNA interference or proteasome inhibition, the stimulatory actions of IGF-I are unmasked and neurons “become” responsive to IGF-I. This interpretation predicts that frataxin promoter activity in neurons will be higher than in other cell types, and that frataxin levels in neurons are controlled by a proteasome-sensitive mechanism, pointing to potential new pathways to explore for therapeutical purposes. Whether this regulatory balance is present in other neuronal types, such as dorsal root ganglia neurons, the primary target of FRDA pathology, remains to be explored. As IGF-I also modulated other mitochondrial proteins such as SOD2 (but not aconitase), the action of IGF-I on frataxin may actually reflect a broader mito-protective effect of this pleiotropic neuroprotective factor.

The combined neuroprotective actions of IGF-I on neurons and astrocytes open the possibility of combat neurodegeneration by enhancing frataxin levels in deficient cells together with potentiation of the neuroprotective properties of astrocytes. The therapeutic potential of IGF-I is reinforced by the positive effects exerted by IGF-I on frataxin in cardiomyocytes, as cardiopathy is frequently present in FRDA patients, and in human astrocytes, indicating that findings using murine models may be translated into clinical practice. The fact that IGF-I protects neurons from frataxin deficiency in a non-cell autonomous fashion also provides new potential targets for therapy by exploring the underlying mechanisms. For instance, IGF-I may promote the release of cytoprotective factors by astrocytes that can rescue both astrocytes and neurons from the absence of frataxin. This possibility is currently under investigation.

Our initial in vivo study indicates that the neuroprotection exerted by IGF-I in vitro may be of therapeutical consequence. Indeed, treatment with IGF-I of moderately ataxic FRDA-like mice (YG8R mice) normalized their motor coordination even though brain frataxin levels remained unchanged. The latter is at odds with our in vitro observation that IGF-I stimulates frataxin in cultured cells. One possibility is that IGF-I cannot stimulate frataxin in mice bearing the mutated frataxin gene because the triplet repeat interferes with its actions on frataxin synthesis. Alternatively, and less likely because the YG8R mice bears the whole promoter region of the frataxin gene, IGF-I may not increase frataxin because the transgene construction lacks the IGF-I-sensitive regulatory region in the frataxin promoter. Direct analysis on human cells from FRDA patients will answer these possibilities. Other possibilities are that the protective effects of IGF-I, as seen in vitro, involve not only a stimulatory effect on frataxin but also other neuroprotective processes that are sufficient to ameliorate the modest functional deficit of YG8R mice. Another possibility is that we cannot detect changes in frataxin levels in YG8R mice treated with IGF-I because of lack of sensitivity of our western blot, as measuring changes in frataxin levels in the brain of YG8R mice is not straightforward [22].

 Although we cannot determine whether IGF-I will be effective in reverting more severe ataxia, as no effective treatments are available yet for this disease, and preliminary studies show beneficial effects of IGF-I in spinocerebellar ataxic patients [7], these results encourage its use in FRDA patients as well. In fact, recent preliminary studies in FRDA patients treated with IGF-I for 1 year show a clear beneficial effect of this neurotrophic factor on disease progression (J Arpa, personal communication).

## Conclusions

These observations indicate that IGF-I exerts cell-specific and context-dependent actions on neurons reinforcing the notion that this growth factor may serve as a therapeutic agent by addressing emerging properties of the diseased brain not present under healthy conditions [1].

## Methods

### Materials

Human IGF-I was purchased from ProSpec-Tany. The protein synthesis inhibitor cycloheximide, the proteasome inhibitor MG-132 (carbobenzoxy-L-leucyl-L-leucyl-L-leucinal) and the phosphatidylinositol 3-phosphate inhibitor (LY294002) were from Calbiochem. The mTOR inhibitor rapamycin and the mRNA transcription inhibitor actinomycin D were from Sigma-Aldrich. Primary antibodies included anti-frataxin (H-155, 1:1000; Santa Cruz Biotechnology), PGC-1 (1:1000; Santa Cruz Biotechnology)*,* Cre Recombinase (1:10000; Novogen), phospho-mTOR (1:2000, Cell Signaling), mTOR (1:2000, Cell Signaling), SOD2 (1:4000, Enzo Life Sci.), and β-actin (1:100000; Sigma). Secondary antibodies were goat anti-rabbit (1:10000) or mouse HRP-coupled (1:10000) both from Bio-Rad.

### Animals

Conditional FRDA mouse mutants of both sexes in which exon 4 of frataxin is loxP-flanked (a kind gift of Michel Koenig, Strasbourg University) congenic (> 10 generations) with C57Bl6 mice, and wild type mice of the same strain were used. Multiplex PCR for mouse genotyping included a common forward primer (P3, 5'−CTG TTT ACC ATG GCT GAG ATC TC−3') and two reverse primers specific for the wild-type (P4, 5'−CCA AGG ATA TAA CAG ACA CCA TT−3') and mutant (P2, 5’-CGC CTC CCC TAC CCG GTA GAA TTC-3’) alleles. Compound FRDA mutants known as YG8R mice [22] were obtained from Jackson Labs (Jax) and bred according to its guidelines. The YG8R mouse is currently considered the best animal model of FRDA as it bears human mutated frataxin without endogenous murine frataxin. Thus, at the molecular level this mouse mimics the disease. However, as is usually the case in transgenic mice, the phenotype only partially resembles the human condition. YG8R mice show very modest ataxia, the neurological hallmark of FRDA. Neuropathology findings, as originally described by the authors [22] are similar to those in human patients but less pronounced. Mice were genotyped by PCR following Jax protocols. Even though recent findings indicate that only female YG8R mice show ataxia we used both males and females (42% males) for these studies as breeding of these mice require large amounts of animals to be produced and in our hands both sexes performed similarly in the rota-rod test. The same gender proportion was kept in control mice. All mice had access to food and water *ad libitum*, were kept under 12:12 hour light/dark conditions and handled according to institutionally approved procedures. YG8R mice treated with subcutaneous IGF-I using Alzet minipumps for 1 month had human IGF-I levels in blood of 22.1 ±1.26 ng/ml (human-specific ELISA from R&D, see [24]). Control C57BL6 mice had undetectable levels

### Cell cultures and transfections

Astroglial cultures with >95% GFAP-positive cells were prepared as described [25]. Postnatal (day 3–4) brains were dissected and immersed in ice-cold Hank’s balance salt solution (Invitrogen). Cortex and hippocampus were removed and mechanically dissociated. The resulting cell suspension was centrifuged and plated in DMEM/F-12 (Invitrogen) with 10% fetal bovine serum (Invitrogen) and 100mg/ml of antibiotic-antimycotic solution (Sigma). After 15–20 days, astrocytes were re-plated at 1,2 × 10^5^ cells/well. Pure cerebellar granule neurons from postnatal mouse cerebellum (>99% β3-tubulin^+^ cells) were obtained as previously described [26]. Briefly, P7 brains were dissected and immersed in ice-cold Hank’s balance salt solution (Invitrogen). Cerebella were removed and first dissociated mechanically, and then enzimatically digested with papain solution (Worthington). Finally, neurons were plated in wells covered with poly-L-lysine in medium with Neurobasal plus B27 (Invitrogen), glutamine, and 25 mM KCl at 4.5 × 105 cells/well. Cardiomyocyte cultures from the cardiac ventricles of neonate mice were prepared as described [27]. In brief, excised hearts were separated into ventricular and atrial tissues, and the ventricles were dissociated by serial enzymatic digestion (5 digestions of 10 minutes each) with 24 mg of collagenase type II (GIBCO) and 50 μg of DNAse (Sigma) in 50 ml of HBSS, using a shaker at 37°C. Myocytes and non-myocytes were separated by pre-plating for 30 minutes onto 10 cm^2^ plates in medium containing 4:1 DMEM high glucose: M199 (GIBCO), 10% horse serum, 5% FCS, and 100 mg/ml antibiotic-antimycotic.

Frataxin deficiency in neurons and astrocytes was elicited following two different systems. First, we used viral transduction using a three-plasmid system as described previously (Dull et al., 1998). The co-transfection system consisted of Mission^®^ shRNA (Sigma-Aldrich), the packaging construct (pCMVΔR-8.74) and the vesicular stomatitis virus G-protein envelope (pMD2.VSV.G). The Mission^®^ shRNAs were: turbo GFP (SHC0003V), non-target shRNA (SHC002) or frataxin shRNA (NM_000144). The transfer vector (5 μg), the envelope (2 μg), and the packaging plasmids (5 μg) were co-transfected using calcium phosphate in human embryonic kidney 293 T cells (6 × 10^6^ cells per dish) cultured in DMEM (Invitrogen, San Diego, CA) with 10% FCS and 1% penicillin/ streptomycin. Lysosomal function was inhibited with cloroquine prior to transfection. The supernatant containing the viral particles was collected, filtered and stored at −80°C. Viral concentration was titrated by cytometry (FACSAria cytometer, BD Biosciences, San Diego, CA). Infection efficiency was ~80% as determined using GFP-expressing viral particles. Frataxin levels were reduced around 50% compared to scrambled-transfected cells. In a second method aimed to produce more drastic frataxin deficiency cells from Lox flanked Fxn transgenic mice were transfected with a Cre recombinase-GFP plasmid using fugene 6 (Roche) or Neurofect (Genlatis) for astrocytes or neurons, respectively. Fugene 6 was used in a 1:3 ratio (DNA:transfection agent) and Neurofect in a 1:6 ratio. Astrocytes were used 2 weeks after transfection and neurons were transfected 1–2 days after plated and used 24 hours later. Plasmids used were peGFPC1-CRE recombinase or pCMV-GFP (control). Before stimulation with 100 nM IGF-I, FBS was removed from the plates. After 24 hours, cells were processed for Western blot or qPCR. When inhibitors were used, FBS was removed 3 hours prior to the addition of the different inhibitors (cycloheximide 1 μg/ml, actinomycin D 2 μg/ml, MG-132 10 μM, rapamycin 100 nM or LY294002 100 μM). Cells were maintained with the inhibitors for 3 hours before adding IGF-I. Treatments were done in duplicate or triplicate in at least 3 independent experiments.

### Cell assays

Cell viability was assessed in neurons nucleoporated (Amaxa) with the peGFPC1-CRE recombinase plasmid and plated over astrocytes. GFP^+^ neurons were scored after 24 hours of IGF-I treatment. Neurons were counted in ten different fields per well at 10×. GFP^+^ cells were related to total cell number determined with DAPI nuclear staining. Cell counts were performed with Metamorph software using the multi wavelength cell scoring module. Generation of ROS was assessed with MitoSOX™ Red reagent from Molecular Probes following the manufacturer’s procedure using the cytometer equipped with a double Argon (488 nm) and Helium-Neon laser (633 nm). Data were collected by using a linear digital signal process. The emission filter used was BP 585/42 for MitosoxRed (FL2). Appropriate values of electronic compensation were adjusted between fluorescence when needed. Debris and duplets were always excluded from the analysis. Data were analyzed with FACSDiva data analysis software (BD Biosciences) and displayed using bi-exponential scaling. Mitochondrial activity was measured using a commercial MTT (3–4,5-dimethylthiazol-2-yl-2,5-dypheniltetrazolium bromide) assay (Roche Diagnostics, Switzerland) as described [28]. Assays were done in triplicate dishes. Production of ROS was also determined by analyzing H_2_O_2_ levels in the cultures as described previously (Brera et al., 2000), with minor modifications. Briefly, the method uses the nonfluorescent cell-permeant compound 2′,7′-dichlorofluorescein diacetate (Invitrogen) which can be oxidized by peroxides to produce the fluorescent compound 2′,7′-dichlorofluorescein. Generation of peroxides was measured in a FLUOstar plate reader (BMG Lab technologies, Offenburg, Germany) at an excitation wavelength of 485 nm and an emission of 520 nm.

### Immunoassays

Animals were perfused transcardially with saline before collection of brain samples for biochemical analysis. Cells or cerebral cortex were homogenized in ice-cold lysis buffer (1% NP-40, 150 mM NaCl, 20 mM Tris pH 7.4 , 10% glycerol, 1 mM Cl2Ca, 1 mM Cl2 Mg, 400 μM sodium orthovanadate, 200μM phenyl methylsulfonyl fluoride (PMSF), 1ug/ml leupeptine, 1ug/ml aprotinin). Western blot was performed as described [29]. Membranes were re-blotted with β-actin as internal standards and to normalize for protein load. The ratio of relative expression was established after subtraction of the background intensity. Levels of the protein under study were expressed relative to protein load in each lane. Species-specific IGF-I ELISA was performed in brain and serum samples as described [24].

### Quantitative PCR

Total RNA was extracted from 1.5 × 10^6 ^cells using illustra RNAspin Mini (GE Healthcare). RNA was reverse transcribed using High Capacity cDNA Reverse Transcription Kit (Applied Biosystems) according to the manufacturer’s instructions. One μl sample of cDNA was amplified using TaqMan probes for frataxin (NM_008044.2) and GAPDH as endogenous control (Applied Biosystems). Each sample was run in triplicate for both frataxin and GAPDH in 20 μl reaction using Taqman Universal PCR Master Mix according to the manufacturer’s instruction (Applied Biosystems). Reactions were performed in an ABIPrism 7000 sequence detector system. Quantitative real time PCR analysis was carried out using the 2(−ΔΔC(T)) method (2-ΔΔCt) as previously described [30]. Results were expressed as relative expression ratios on the basis of group means for target frataxin transcript versus reference GAPDH transcript.

### Rota-rod test

Motor coordination in mutant mice was assessed with the rota-rod test as described previously [3] using the protocol of the authors that developed this mouse [22] because our former protocol did not detect any ataxia in these mice (not shown). The protocol consists of 4 trials of 5 minutes each with a 10 minutes rest between each trial. No other sensorimotor parameters were measured.

### Statistics

Statistical analyses were performed with a t test when comparing two groups and a two-way ANOVA followed by a t-test when comparing multiple groups. Statistical significance was established at p<0.05. Results shown are mean ± SEM.

## Competing interests

ITA holds shares of a company devoted to the use of IGF-I as a neuroprotectant.

## Authors’ contributions

CF conducted and designed experiments, analyzed the results and wrote part of the manuscript. SF conducted and designed experiments. ITA designed the study, analyzed the data and wrote the manuscript. All authors read and approved the final manuscript.
